# Opportunities and challenges in the nanoparticles for nucleic acid therapeutics: the first approval of an RNAi nanoparticle for treatment of a rare disease

**DOI:** 10.1093/nsr/nwz009

**Published:** 2019-02-05

**Authors:** Xiawei Wei, Yuquan Wei

**Affiliations:** Laboratory of Aging Research and Nanotoxicology, State Key Laboratory of Biotherapy, National Clinical Research Center for Geriatrics, West China Hospital, Sichuan University, China

Nanoparticle-based nucleic acid therapeutics have drawn considerable attention over recent decades. By using terms such as ‘nanoparticle’ and ‘gene delivery’ in a PubMed search, we found that 10 years ago throughout 2008 there were only 725 such papers published; however, in 2017, over 4700 papers were published with the same keywords. The dramatic increase in the number of publications indicates that the field of nanoparticle-based nucleic acid therapeutics, which is a complicated one underpinned by the development of numerous related fields, such as pharmaceutics, material sciences, immunology and cell biology, is thriving.

After the devotion of time and energy for decades, a breakthrough for nanoparticle-based nucleic acid therapeutics finally came with the news released on 12 August 2018 that the first lipid complex containing small interfering RNA (siRNA) had been approved by the US Food and Drug Administration (FDA) for the treatment of a rare disease [1]. The approved lipid complex injection, named ONPATTRO™ (patisiran), is for the treatment of the polyneuropathy of hereditary transthyretin-mediated amyloidosis, and was reported as a ‘historic approval’ and an ‘important milestone’ for the treatment of a rare disease, as well as for nanoparticle-based nucleic acid therapeutics.

**Figure 1.**
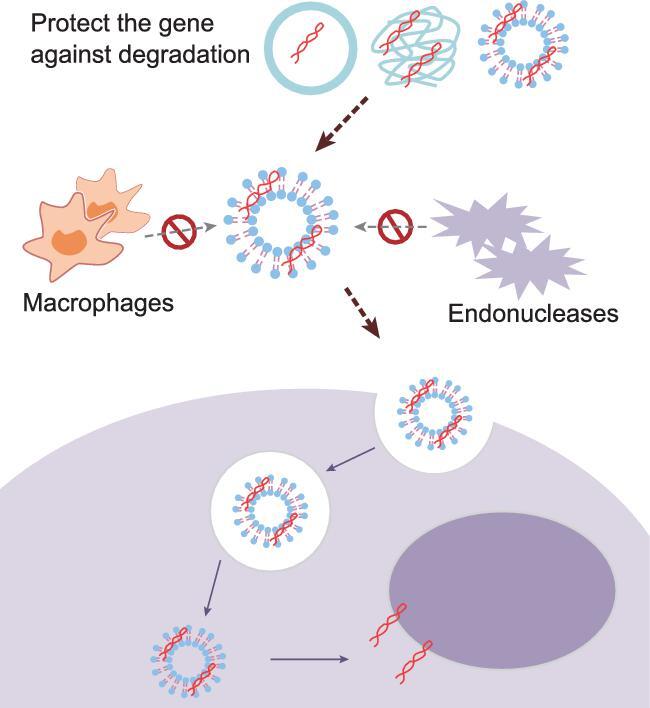
The ideal nanoparticle in a nucleic acid delivery system should protect the gene against degradation by nucleases and internalize the plasma membrane, while escaping from the endosomal compartment. Subsequently, the nanodelivery system might unpackage the gene at some point with no detrimental effects, and the nanoparticle should also be less immunogenic and low in cost [2]. Figure is redrawn from Yin *et al*. [2].

The characteristics of the nucleic acid delivery system play key roles in the development of a successful nanoscale therapeutic [2]. Researchers’ enthusiasm for seeking better non-viral vectors comes from the fact that they have several advantages over viral vectors, such as variability in design and ease of large-scale production. Ideal nanoparticles are supposed to have distinctive features (Fig. 1) and should: i) protect a gene against degradation by a nuclease; ii) internalize the plasma membrane and escape from the endosomal compartment; iii) unpackage the gene at some point and have no detrimental effects; iv) be less immunogenic; and v) be low in cost. Surface-charged cationic nanoparticles, such as cationic liposomes and cationic polymers, are useful for nucleic acid delivery due to their electronic interactions with anionic nucleic acids, allowing the formation of drug-loaded complexes. However, positively charged nanoparticles have had limited success in pre-clinical/clinical applications, particularly due to safety issues.

A recently published review traced back through clinical trials concerned with cationic nanoparticles and nucleic acid delivery over the years [3]. Until 2015, no more than 30 clinical trials on cationic nanoparticle-mediated gene delivery had been reported, although thousands of basic research papers concerning cationic nanoparticles are published each year. Furthermore, among the reported cases of clinical studies, most of them failed at the phase I stage and some were even discontinued due to severe nanoparticle-related side effects. These results remind researchers to look into the fundamental mechanisms of cationic particles that induce toxicity, rather than developing so-called ‘multifunctional’ or ‘sophisticated’ novel particles without considering their possible future application in human bodies.

It is not surprising that cationic nanoparticles cause side effects when injected into the human body. The cell toxicity and inflammatory responses induced by cationic particles have been observed in many studies; however, the fundamental mechanisms of how cationic nanoparticles induce cell death and inflammation have only come to light in recent years. Wei *et al.* discovered that cationic nanoparticles induce acute cell necrosis after minutes of incubation with lung epithelial cells, which is due to the impairment of Na/K-ATPase on the cell membrane mediated by specific binding of cationic nanoparticles to the ouabain binding site on the Na/K-ATPase [4]. Moreover, the necrotic cells could release a group of intracellular substances known as Damage-Associated Molecular Patterns, such as mitochondrial DNA and formyl peptide, to stimulate neutrophils and induce subsequent inflammation through the TLR9-Myd88 pathway. This work demonstrated the key role of the surface charge of a nanoparticle in inducing cell necrosis, due to positively charged nanoparticles having a higher affinity (lower binding energy) for the ouabain binding site on Na/K-ATPase and thus causing the acute cell necrosis, which is also responsible for the inflammatory toxicity. Although every kind of nanoparticle has a unique nanotoxicology profile based on its inherent properties and *in vivo* behaviors [5], here we suggest that, for the design of safer and better cationic nanoparticles, consideration should be given to how to reduce or hinder the positive surface charge of the particle, e.g. by finding a balance by decorating the cationic particle with an anionic targeting ligand (such as hyaluronan or folate) [6]. Notably, the successful approval of ONPATTRO™ might be largely due to the unique design of its lipid-based nanoparticle, which is described as being formed from five main components. Although the clinical trial of ONPATTRO™ indicated some of the typical side effects associated with nanoparticle-mediated gene delivery, such as flushing, back pain, nausea and abdominal pain, the side effects could be controlled at an level.

With the approval of the first lipid particle-based nucleotide nanomedicine, it is believed that more nanoparticle-based nucleic acid therapeutics will enter the market. Several aspects could be addressed by scientists attempting to develop a nucleic acid therapeutic candidates. Firstly, more attention could be paid to the specific indication for the therapeutics, i.e. a major disease like cancer or a rare disease with limited treatment options might be a good option. Secondly, investigation of siRNA-based nanotherapeutics should be encouraged for its successful approval by the FDA. Thirdly, emerging new technologies such as gene editing with a clustered regularly interspaced short palindromic repeat-Cas9 system and aptamer selection could be used in the next generation of gene delivery systems for increase therapeutic efficacy [7,8]. Fourthly, we should continue to seek the safer nanomaterials with good biocompatibility, less toxicity, as well as better targeting properties, i.e. cell/tissue-targeted delivery that can be achieved by modifying nanoparticles with specific adaptors, ligands or antibodies [9]. Moreover, the large-scaled production protocol for nanoparticles might also influence their efficacy and safety issues, and should be addressed.

Finally, we would like to thank the members in the editorial office of NSR for giving us the opportunity to share our opinion on nanoparticle-based nucleic acid therapeutics with you all.
